# Characterization of two in vivo challenge models to measure functional activity of monoclonal antibodies to *Plasmodium falciparum* circumsporozoite protein

**DOI:** 10.1186/s12936-020-03181-0

**Published:** 2020-03-17

**Authors:** Rama Raghunandan, Bryan T. Mayer, Yevel Flores-Garcia, Monica W. Gerber, Raphael Gottardo, Hugo Jhun, Sonia M. Herrera, Daniel W. Perez-Ramos, Emily Locke, C. Richter King, Fidel Zavala

**Affiliations:** 1 PATH’s Malaria Vaccine Initiative, 455 Massachusetts Avenue, NW, Suite 1000, Washington, DC 20001 USA; 2 Department of Molecular Microbiology and Immunology, Malaria Research Institute, Johns Hopkins Bloomberg School of Public Health, Baltimore, MD USA; 3 Vaccine and Infectious Disease Division, Fred Hutchison Cancer Research Center, Seattle, WA 98109 USA; 4 Public Health Sciences Division, Fred Hutchinson Cancer Research Center, Seattle, WA 98109 USA; 5 Present Address: Vaccine Research and Development, Pfizer, Pearl River, NY 10965 USA

**Keywords:** Malaria, Transgenic parasite, Bioluminescence, Monoclonal antibodies, Functional activity, Circumsporozoite protein (CSP), *P. falciparum*, *P. berghei*

## Abstract

**Background:**

New strategies are needed to reduce the incidence of malaria, and promising approaches include the development of vaccines and monoclonal antibodies (mAbs) that target the circumsporozoite protein (CSP). To select the best candidates and speed development, it is essential to standardize preclinical assays to measure the potency of such interventions in animal models.

**Methods:**

Two assay configurations were studied using transgenic *Plasmodium berghei* expressing *Plasmodium falciparum* full-length circumsporozoite protein. The assays measured (1) reduction in parasite infection of the liver (liver burden) following an intravenous (i.v) administration of sporozoites and (2) protection from parasitaemia following mosquito bite challenge. Two human CSP mAbs, AB311 and AB317, were compared for their ability to inhibit infection. Multiple independent experiments were conducted to define assay variability and resultant impact on the ability to discriminate differences in mAb functional activity.

**Results:**

Overall, the assays produced highly consistent results in that all individual experiments showed greater functional activity for AB317 compared to AB311 as calculated by the dose required for 50% inhibition (ID50) as well as the serum concentration required for 50% inhibition (IC50). The data were then used to model experimental designs with adequate statistical power to rigorously screen, compare, and rank order novel anti-CSP mAbs.

**Conclusion:**

The results indicate that in vivo assays described here can provide reliable information for comparing the functional activity of mAbs. The results also provide guidance regarding selection of the appropriate experimental design, dose selection, and group sizes.

## Background

Malaria remains a global health emergency, with an estimated 228 million cases and over 400,000 deaths occurring worldwide in 2018 [1]. While control measures, such as insecticide-treated nets, sensitive diagnostics tools and effective case management, have helped reduce the number of cases and deaths, progress has stalled in recent years [1]. New tools, including novel drugs and vaccines, are urgently needed, particularly in the areas of highest endemicity.

Recently, pilot implementation programmes to deliver the RTS,S vaccine in select areas of three African countries began [2]. RTS,S is effective in young children, but efficacy wanes over time [3]. RTS,S elicits responses to the circumsporozoite protein (CSP), the major surface protein on the infecting sporozoite [4]. Antibodies against the NANP repeat regions of CSP induced by RTS,S have been linked to its protective efficacy and these wane after vaccination with kinetics that mimic the decline in efficacy [5]. A second vaccine that targets CSP, R21, has also shown efficacy in nonclinical studies [6] and recently in clinical testing [7]. Radiation attenuated sporozoites, PfSPZ, administered by intravenous (i.v.) injection have shown efficacy in some populations [8] and induce both antibody responses to CSP and cellular responses to multiple antigens expressed during the liver stage [8].

Monoclonal antibodies (mAbs) to CSP have been proposed as new infection-blocking interventions [9–12], and protection by mAbs can be expected to wane with the half-life of the antibody in serum. While altering the sequence of mAb Fc regions has been used to extend serum half-life of mAbs [13], an important additional approach for improving the durability of protection is to identify mAbs effective at lower concentrations. Identification and development of such potent antibodies will require assays that reliably measure their functional activity.

New molecular tools have aided identification and development of human mAbs that bind to CSP. [11, 14, 15] from humans naturally exposed to *Plasmodium falciparum* [10], volunteers vaccinated with RTS,S [16], or from whole sporozoites [17]. The functional activity of these antibodies varies, with potent antibodies binding to the NANP repeat region [10, 11, 16] and to a region just upstream of the repeat domain termed the bridging peptide or junctional region [17, 18].

The current study focuses on the use of two in vivo models of *Plasmodium* sporozoite infection to measure the functional activity of anti-PfCSP mAbs. *Plasmodium* parasites are highly host species specific, with *Plasmodium berghei* efficiently infecting mice while *P. falciparum* fails to do so. *Plasmodium berghei* parasites can be engineered to replace the native CSP with *P. falciparum* parasites [19]. These parasites can be further engineered to express luciferase upon liver infection, allowing simple measurement of infection by measuring luciferase-induced luminescence from infected liver cells. Parasites can be administered by i.v. injection and mosquito bite challenge. Functional antibody activity can be measured either as the ability to inhibit liver infection measuring a luminescence endpoint or to block infection entirely as monitored by the appearance of parasitaemia in the blood. Methods used to conduct these assays were recently described [20]. An alternative model has also been reported in which mice have been engineered to contain human hepatocytes which allows infection by *P. falciparum* sporozoites [21, 22].

The experimental conduct of in vivo protection assays is inherently complex, requiring the consistent execution of multiple steps including preparation of the infectious challenge dose as either isolated sporozoites or infected mosquitoes, consistent delivery of the protective mAb in animal models, as well as monitoring of the infection endpoint. As the overall goal for such assays is to reliably identify antibodies with high potency, results were compared for multiple experiments conducted with the same methods and reagents. To assess each assay’s utility in selecting the most potent mAbs results were collected on intra- and inter- experiment consistency using two mAbs with high protective potency [16]. The results demonstrate that consistent information on mAb potency can be obtained using the procedures applied and provide guidance for the selection of superior mAbs as clinical candidates. The results may also be extendable to the testing of improved CSP targeted vaccines.

## Methods

### Materials

*Animals:* Female mice C57Bl/6 6–7 weeks of age were purchased from Charles River Laboratories, Frederick MD USA. All studies were performed under the protocol MOI7H369, approved by the ACUC at JHU.

*Antibodies:* AB311 and AB317 are human immunoglobulin G 1 (IgG1) mAbs isolated from an experimental clinical trial of RTS,S, MAL071 [23] clinical trial and both mAbs bind to NANP repeats [16]; mAb1245 is also a human IgG1 mAb, isolated from a Kymab mouse, and binds to a *P. falciparum* ookinete protein Pfs25, and thus is used as a negative isotype control [24]. All mAbs were expressed by transient transduction 0.5 L TunaCHO cultures followed by protein A purification at Lake Pharma Inc. Belmont, CA.

*Parasites:* Transgenic sporozoites in *P. berghei* expressing *P. falciparum* CSP, green fluorescent protein and luciferase reporter gene, used in all studies, has been previously described [20] and methods for parasite preparation have been described in detail [20]. Briefly, 5-day old adult *Anopheles* *stephensi* mosquitoes were allowed to feed on mice carrying 1 to 2% transgenic parasites. Twenty to 22 days post murine blood meal, transgenic sporozoites were collected from salivary glands and used within 60 min. for i.v. infection for liver burden studies. For parasitaemia studies, infectious mosquitoes were used directly.

### Reduction in parasite liver burden assay

As previously described [20], mice were injected intravenously in the tail-vein with test and control antibodies at the indicated concentration in PBS (200 µL). Challenge with transgenic sporozoites occured 16 h following mAb administration. Forty-two hours after parasite challenge, parasite load in the liver was measured by bioluminescence in an in vivo imaging system (IVIS Spectrum, Perkin Elmer). Mice were injected intraperitoneally with 100 µL of d-luciferin (30 mg/mL) and immediately anesthetized with isoflurane for 5 min prior to IVIS. Groups of five anesthetized mice were placed in the imager and the radiance measurements recorded by the live imager software, version 4.5.1. The total flux reading for each mouse was recorded individually. Background reading was verified for each study with two naïve mice that received only the d-luciferin substrate; these are reported in Table 1 as naïve controls.

**Table 1:** Assay consistency across studies

Dose (µg)	Log 10 flux values for studies 1 to 7 using specified mAb															
	1—AB311		2—AB311		3—AB311		4—AB311		4—AB1245		5—AB311		6—AB311		7—AB311	
	Mean	SD	Mean	SD	Mean	SD	Mean	SD	Mean	SD	Mean	SD	Mean	SD	Mean	SD
600	Not applicable (N/A)						5.38	0.05	7.40	0.11	5.31	0.26	5.40	0.13	5.31	0.05
300	5.83	0.24	5.84	0.22	5.84	0.25	5.56	0.17	7.43	0.16	5.82	0.18	5.77	0.10	5.58	0.17
100	6.41	0.20	6.57	0.17	6.53	0.35	6.46	0.21	7.32	0.08	6.01	0.21	6.25	0.23	6.16	0.15
30	7.06	0.11	7.05	0.12	N/A		7.03	0.11	7.47	0.06	6.84	0.12	6.52	0.15	6.64	0.16
10											6.95	0.12				
Untreated	7.62	0.06	7.48	0.05	7.46	0.08	7.47	0.15	7.47	0.15	7.22	0.10	7.25	0.06	7.20	0.15
Naïve	5.09	0.09	5.11	0.03	5.03	0.18	5.08	0.13	5.07	0.12	5.24	0.03	5.25	0.02	5.25	0.03

Studies 1, 2, 3 did not include a 600-µg group. Only study five had a 10-µg group. Individual mouse total flux values were used to calculate geometric mean for each dose group

*mAb* monoclonal antibody, *SD* standard deviation as estimated using a random effects model as described in methods

### Protection from mosquito bite challenge assay

As previously described [20], *An.* *stephensi* mosquitoes were fed on mice infected with chimeric *P. berghei* parasites encoding full-length *P. falciparum* CSP. In order to determine the proportion infected, 19–20 days after blood feeding on mice, a few mosquitoes were dissected to determine whether sporozoites were present in salivary glands.

Sixteen hours after administration of mAbs, C57Bl/6 mice were anesthetized with 2% Avertin and exposed to five infected mosquitoes for ~ 10 min. The number of mosquitoes that fed on blood was determined by observation of a red abdomen. Days 4–12 post challenge, blood smears from the tip of the mouse’s tail were collected, stained with 10% Giemsa, and examined by light microscopy to determine the presence of blood stage parasites.

### Monoclonal antibody serum pharmacokinetics

Blood was collected from mice via retro-orbital sinus one hour prior to challenge (fifteen hours after prophylaxis). Blood was processed to serum using standard methods and stored at 4 °C until assayed. Three-fold dilution series against Nunc MaxiSorp™ plates (ThermoFisher Scientific) coated with 5 ng/mL recombinant CSP were assayed in standard ELISA assays using HRP-conjugated Goat anti-Human IgG (H + L) secondary antibody (Jackson ImmunoResearch) and ABTS peroxidase substrate (KPL). Human IgG concentration in serum was determined by comparison to mAb standard curves.

### Analysing parasite liver burden assay repeatability

Overall inter- and intra- study liver burden assay variability was assessed by analysing the standard deviations (SD) of log_10_ total flux estimated using random effects models with log_10_ flux as the outcome and a random intercept specified for each study. This model was fit to the naïve, infected control data to assess both assay and infectivity variability. For treated animals (mice receiving potent mAbs), a fixed effect for dose was also included to control for treatment effect (i.e., a linear mixed effects model was fit). From these models, inter-experiment variance was estimated as the variance of the random effect and intra-experiment variance was estimated as the residual variance. Estimated standard deviations of log_10_ flux were transformed back (unlogged) to be interpreted as fold-change deviations.

### Dose–response modelling and estimating ID50 and IC50

The relationships between dose or circulating mAb and assay outcomes were modelled using four-parameter logistic (4PL) models with the following functional form: y = L+(U − L)/(1 + (*x*/ID50)^h^).

Where:

- L is the minimum value of the assay reached as the protective dose or circulating mAb levels increase toward infinity, or measured in negative control animals (i.e., lower limit or lower saturation);
- U is the maximum value of the assay for dose or circulating mAb of 0, or measured in naïve, infected animals (i.e., upper limit or upper saturation);
- ID50 is the dose (IC50 for circulating antibody concentration) where the outcome is 50% reduced relative to U and L (the point of inflection); and
- h is the Hill slope determining steepness in the linear section of the curve.

For the liver burden assay, the outcome modelled was log_10_ flux. The upper limit was estimated by including the naïve, infected controls as equivalent to a dose or circulating mAb concentration of 0 in the given experiment. Thus, each dose–response curve within an experiment was adjusted for the infectivity of the challenge strain for that study. The lower limit was fixed to 5 log_10_ flux based on all of the negative control data. The ID50 (or IC50) then represents the dose (or circulating mAb concentration) at which flux is 50% reduced relative to the upper and lower log_10_ flux for that study.

For the protection from parasitaemia assay, the lower and upper limits were fixed ranging from 0 to 100% infected. As only the ID50 or IC50 and the slope were fitted, this is a 2PL model. Here, the ID50 (or IC50) represents the dose (or circulating mAb concentration) where animals have a 50% probability of infection (or are 50% protected).

### Statistical methods for comparing liver burden reduction

To compare mAb potencies using the liver burden assays, 4PL models were fit to both mAbs within a study and the estimated ID50s and IC50s (on the log-scale) were compared using a *t* test. Comparisons were performed by pooling the data across the studies where both mAbs were tested.

### Statistical methods for protection from parasitaemia

Protection from parasitaemia was determined by either 1) the proportion of uninfected animals remaining after the observation period post-challenge, or 2) the observed infection day following challenge. As the liver burden assay established higher potency of AB317 compared to AB311, all statistical comparisons were one-sided testing for superiority of AB317 over AB311. Within a study, at a single dose, protection was compared using Barnard’s exact test (Z-pooled method, [25]) for each dose with at least partial protection observed in any study (100 μg and 300 μg). Survival curves were also fitted for each experiment, grouping by antibody and dose. To test the null hypothesis that there were no differences in survival time between mAbs, a log-rank test using the exact conditional distribution with the Hothorn-Lausen tie-method [26] was conducted by dose group.

Two approaches were used to compare protection between the antibodies across all of the doses: (1) comparison of ID50s or IC50s (on the log-scale, one-sided) from fitted 2PL models with a common Hill slope for both mAbs using a z-test; and (2) testing for a significant odds ratio between mAbs using a logistic regression with log dose and mAb as predictor.

The 2PL model and logistic regression including log dose are equivalent models if a common Hill slope for both mAbs is fitted in the 2PL model or an interaction term between mAb and log dose is specified for the logistic regression. For the logistic regression, a small sample size correction (Firth-correction) was used to robustly estimate the odds ratio and one-sided 95% confidence intervals through profiling [27]. A one-sided test was then performed for AB317 superior potency (odd ratio < 1) by comparing the upper bound of the CI to 1. Effectively, the logistic regression approach represents a strategy to accommodate for the small sample size constraints of these data by assuming differences in potency are represented strictly by shifts in the ID50 or IC50 and not by steeper or shallower curves.

### Power analysis and sample size determination for study design

Power calculations were performed under the framework that candidate mAbs would be compared to AB311 as a reference in a single dose study for both the liver burden and parasitaemia assays. For the liver burden assay, it was assumed that the chosen dose would represent the ID50 of the reference mAb (AB311) and the candidate mAb would be tested against the reference using a t-test comparing log_10_ flux. The power for the t-test comparison was then calculated by varying effect size and a sample size. Effect size was determined based on a range of theoretical ratios of flux between mAbs (differences in log_10_ flux) and variation measured within the study. The range of flux ratios represents potential increasing potencies for candidate mAbs. Three levels of variation were chosen using the range of standard deviations from the studies to represent low, average, and high variability scenarios. Sample size varied from 5 to 12 per group to represent an experimentally practical range for total mice within a single study. Tests were two-sided with an alpha = 0.05 (5% false positive rate).

To calculate power for the protection from parasitaemia assay, a single dose of 300 µg was chosen to optimally incorporate the empirical data and the Barnard’s exact test was then used to compare protection, matching the data analysis. Potential candidate mAbs were considered with potencies ranging from full protection (100% protection) to equivalent reference AB311 protection at 300 µg (averaged protection across the studies). Power was then calculated across a sample size of 6–12 animals. Tests were one-sided for superiority of the candidate mAb with an alpha = 0.05.

For additional interpretation in the power analysis, ID50s were also estimated from the assay endpoints for the candidate mAbs across the range of tested potencies. For the liver burden assay, this was done by estimating a log_10_ flux based on the fold reduction at the given dose and then deriving the ID50 from the 4PL model using the upper bound (7.22 log_10_ flux) and Hill slope [1] parameters estimated for AB317, a proxy for potential candidate mAbs. Similar for the protection from parasitaemia analysis, the ID50 was derived from the 2PL model using the protection estimate at 300 µg and a fixed Hill slope (common estimate for both AB311 and AB317). Once the candidate mAb ID50 was estimated, the ratio was taken relative to the mean AB311 ID50 by assay.

### Statistical software

Statistical analysis was performed using the R programming language [28] Linear mixed effects models for repeatability analysis were fit using the lme4 package [29]. The model fitting for 4PL and 2PL models and the ID50 and IC50 comparisons were performed using the drc package [30]. The log-rank test was implemented with the coin package [31]. Comparisons and power calculations using Barnard’s exact test (Z-pooled method) were performed using the Exact package [32]. Visualizations were generated using the ggplot2 package [33].

## Results

### Inter-assay consistency in reduction in parasite liver burden studies

Two human active mAbs were tested, AB311 and AB317, that bind to the NANP repeats of *P. falciparum* CSP. These mAbs have previously been shown to bind with high affinity and have in vivo functional activity at a single tested dose [16]. AB317 has also been previously tested across a range of doses (30–300 µg) [20] and this guided the selection of dose levels for the present study. The mAb AB1245 [24] that binds to ookinete antigen Pfs25, was used as an IgG1 isotype matched negative control. The main features of the in vivo functional assays here have been described [34] and experimental details of the assays, including the use the parasites expressing luciferase induced bioluminescence as a measure of liver burden, have been recently reported [20].

To determine the consistency of the reduction in parasite liver burden assay, seven independent experiments were conducted on separate days with different preparations of infectious sporozoites. AB311 was delivered i.v. in a uniform volume of 200 µL at indicated doses, while the sporozoite challenge was administered i.v. post mAb delivery. In all seven studies, mice that received AB311 showed a dose–dependent reduction of transgenic sporozoite infection in liver as measured by the total flux of bioluminescence (Table 1). There was no reduction in transgenic sporozoite infection in mice that received negative control AB1245 (Table 1). The inter-assay consistency results are shown in Table 1. In mice receiving no antibody treatment (untreated infected), the total flux measures were consistent across studies in overall level (range of log_10_ flux 6.95 to 7.62). Using random effects models to estimate variance of log_10_ flux, inter- and intra-assay variation were similar and limited among the untreated, infected mice (estimated standard deviations of 0.16 and 0.10 log_10_ flux or 1.17 and 1.11-fold changes, respectively). These results demonstrate very consistent levels of infectious sporozoites delivery, implying good reproducibility in the preparation, handling, and infectivity of sporozoite batches. Consistency in total level of infection was also seen across experiments for treated groups receiving AB311 (Table 1). Using linear, mixed effects models adjusting for dose variation, the estimated inter-assay variation for treated animals was similar to the inter-assay variation for the infected controls (standard deviation of 0.13 log_10_ flux or 1.14-fold). For intra-assay variation, the standard deviation for log_10_ flux within all groups receiving AB311 ranged from a high of 0.35 to a low of 0.05 across experiments (Table 1), with a model-estimated standard deviation of 0.20 log_10_ flux (1.22-fold). Taken together, the consistency of total flux data for the treatment groups also implies reproducible handling and delivery of mAbs as well as consistent measurement of liver infection by bioluminescence.

To set the background level of luminescence, two naïve mice were used in each study that did not receive any challenge parasite or antibodies, but received only the substrate D-Luciferin. A level of 5.03–5.25 log_10_ total flux was observed across experiments (Table 1). This sets the lower limit, or minimum log_10_ total flux, that can be measured for the liver burden assay. The 600-µg AB311 dose group achieved the log_10_ flux value of 5.40, indicating nearly complete inhibition.

**Fig. 1:**
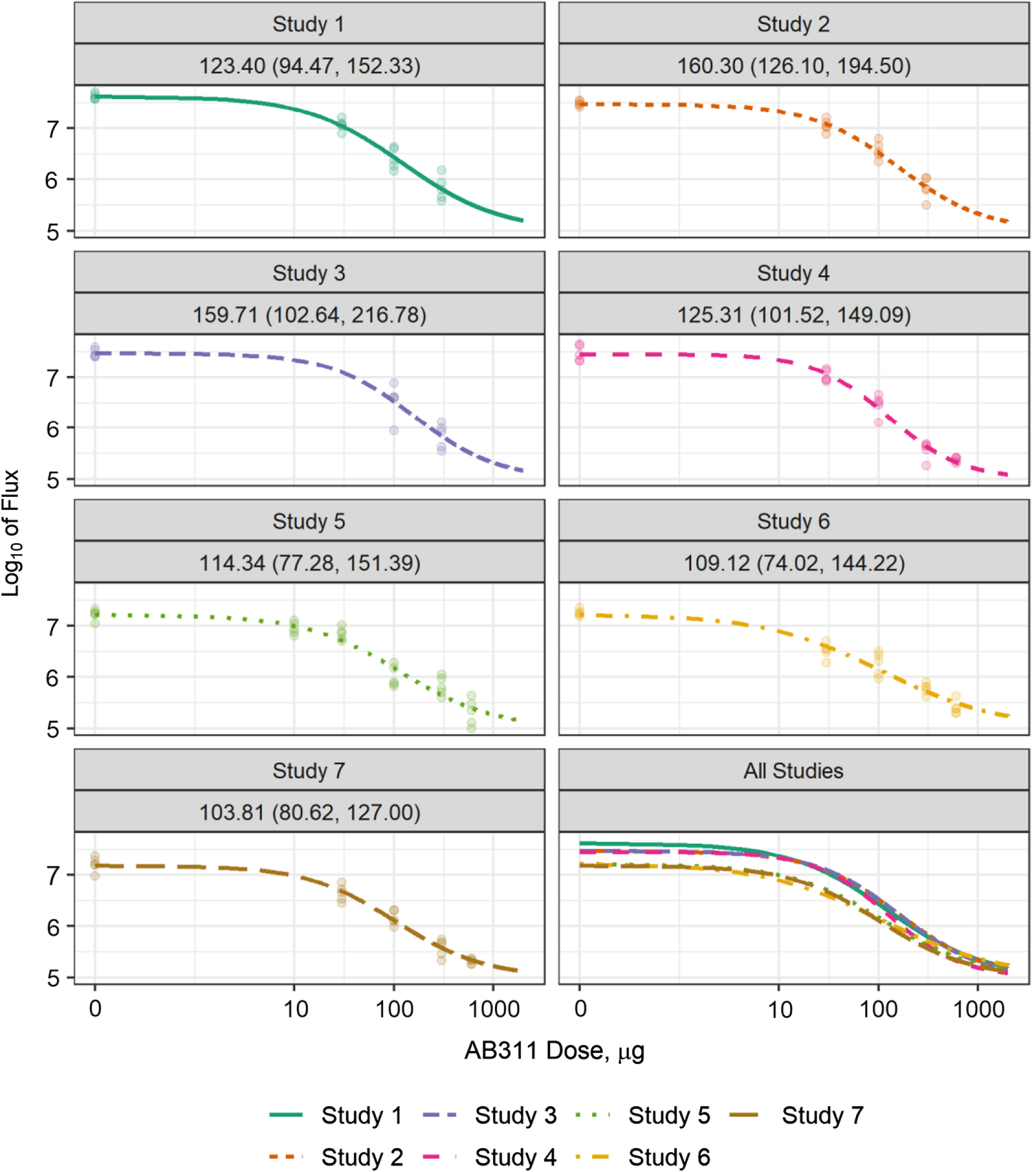
Dose-dependent changes in inhibition of parasite liver burden from seven independent studies. Dose *versus* log_10_ total flux achieved in mice receiving 600, 300, 100, 30, and 0 µg doses of AB311 is shown. Curves represent the dose–response relationship estimated using 4PL models for each experiment and points represent the observed flux for each mouse. Mean and 95% CI for estimated ID50s, the dose by which liver burden is reduced 50% between the upper and lower bounds of measurement, are shown for each study

Consistency of mAb performance of parasite liver burden assay was assessed by comparing ID50 estimates. The ID50 represents the dose at which there is 50% reduction in log_10_ flux between the upper and lower limits of the liver burden measurements. The ID50 was modelled using a four-parameter logistic regression analysis of reduction in log_10_ flux for different dose levels (Fig. 1). In the seven studies, the estimated ID50 for AB311 ranged from 103 to 160 µg with overlapping 95% confidence intervals among the experiments. These results show that for a single antibody, the reduction in liver burden assay can be performed in a highly reproducible fashion and that results are comparable across studies.

### Comparison of functional activity of two mAbs using reduction in parasite liver burden assay

**Fig. 2:**
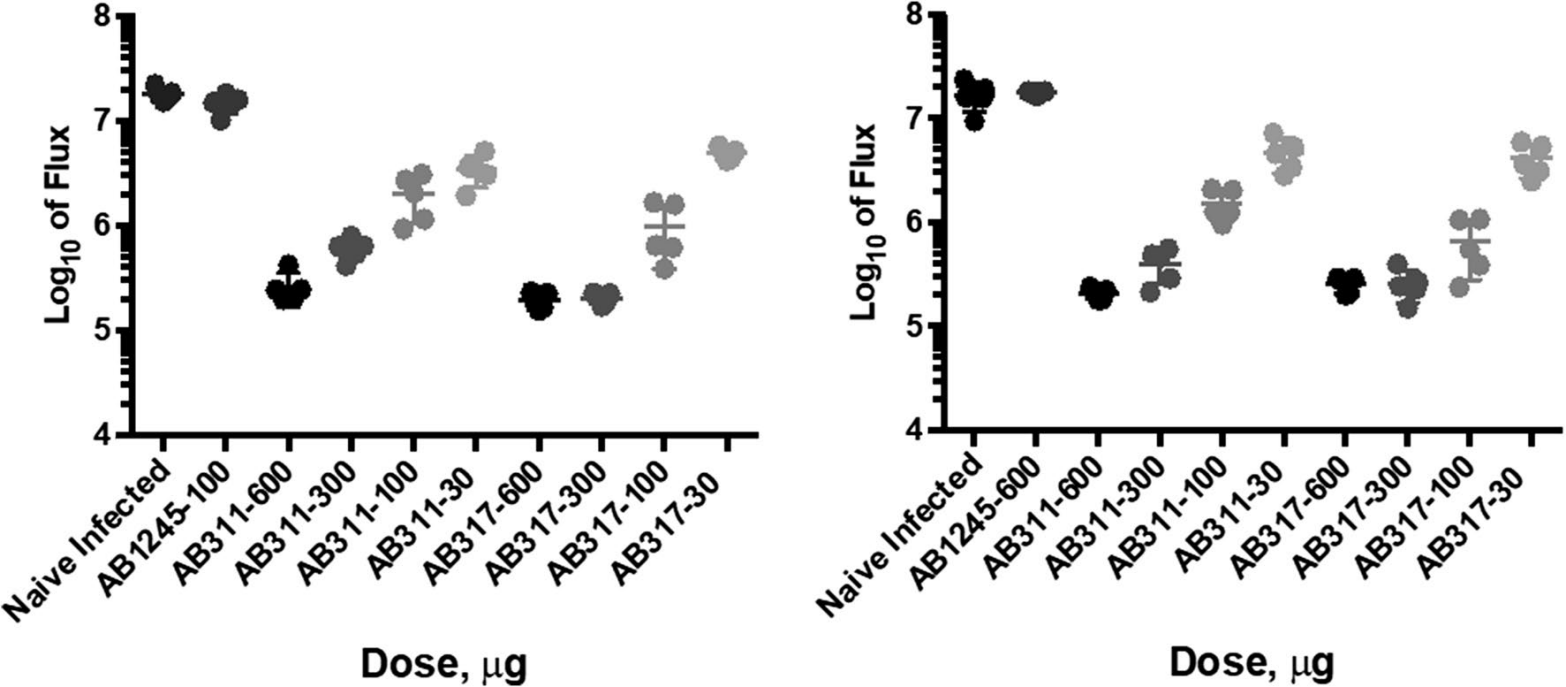
Comparing reduction in parasite liver burden in two studies by mAb: Mice (N = 5) were dosed with AB311 or AB317 at 600, 300, 100 and 30 µg. Each study also had five naïve, infected mice (no mAb administered) and five mice receiving mAb1245 at 600 µg (control with no functional activity). Transgenic sporozoite challenge was administered at 16 h post administration of mAb. Points denote flux measured for each animal, and line represents the average from 5 individual mice per group. Groups containing naïve mice that do not receive any treatment and mice that receive a non-functional mAb (AB1245) are shown for both experiments

An important application of the liver burden assay is to identify mAbs with higher potency compared to a control. To model such a test, AB311 and AB317 were compared in the same study. Mice were dosed with AB311 or AB317 and challenged with transgenic sporozoites using identical assay conditions. Two distinct experiments are shown in Fig. 2. Control groups that received no mAb treatment had a mean log_10_ flux of 7.2 in both studies. The assay standard deviation within all groups receiving treatments was similar to that shown in Table 1, ranging from a minimum of 0.05 to maximum of 0.28 (Additional file 1: Table S1).

To compare potency, differences in ID50s were tested comparing AB311 and AB317 using the 4PL model (Table 2). Compared to AB311, the ID50 for AB317 was lower by 1.5-fold (P = 0.07) and 1.6-fold (P = 0.04) for each experiment, and 1.6-fold lower (P < 0.01) pooling the data across both experiments.

**Table 2:** Comparison of Anti-CSP mAbs in reduction in parasite liver burden

Study number	ID50				IC50			
	AB311 (µg/mL)	AB317 (µg/mL)	Ratio	P value	AB311 (µg/mL)	AB317 (µg/mL)	Ratio	P value
6	109 (74, 144)	74 (59, 87)	1.5	0.07	32 (21, 44)	22 (17, 28)	1.4	0.15
7	104 (80, 127)	64 (44, 83)	1.6	0.04	26 (19, 32)	21 (15, 28)	1.2	0.39
Pooled	107 (85, 128)	69 (58, 80)	1.6	< 0.01	29 (22, 35)	22 (18, 26)	1.3	0.11

ID 50 and IC50 values and 95% confidence intervals (parentheses) and the fold difference (ratio) between antibodies. ID50 results are reported in µg and IC50 reported in µg/mL. All group sizes were five per dose

**Fig. 3:**
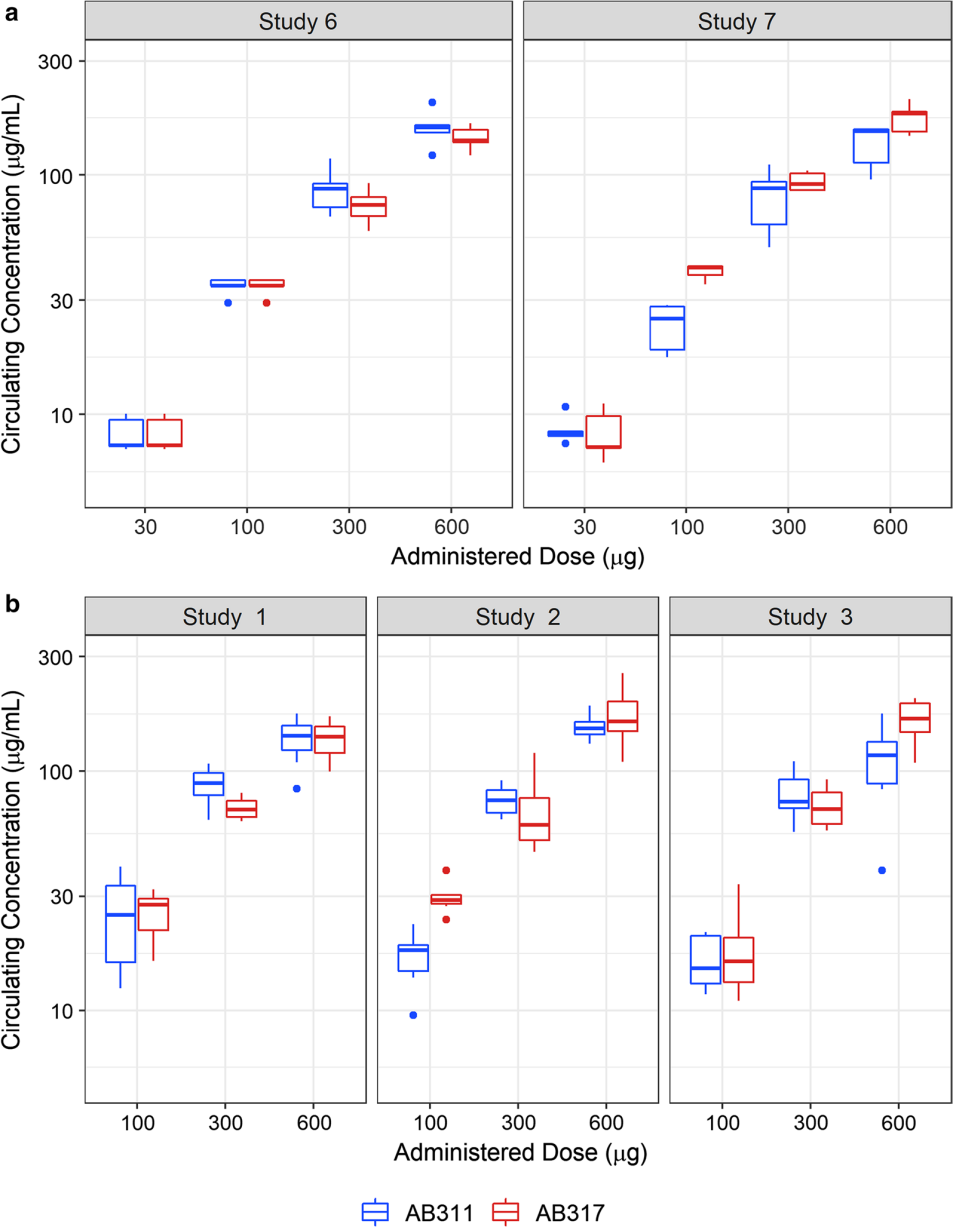
Administered dose versus circulating serum antibodies in reduction in parasite liver burden assay (**a**) and from protection from parasitaemia assay (**b**): serum samples from an hour pre-challenge blood draw from all mice that received AB311 or AB317 were analysed (n = 5 in all groups). In anti-CSP ELISA and the circulating serum antibody levels measured in µg and plotted against the administered dose. The mid-line of the box denotes the median and the ends of the box denote the 25th and 75th percentiles. The whiskers denote the most extreme data points that are no more than 1.5 times the interquartile range (i.e., height of the box). Study numbers shown are consistent with Tables 2 and 3

Circulating mAb serum concentrations 1 h prior to the time of challenge were measured using a CSP ELISA (Fig. 3a). IC50 was determined using 4PL model and results are reported in Table 2. While there was a trend toward higher potency of AB317 compared to AB311, differences in IC50s were not statistically significant between the two mAbs within either experiment or when pooling the data across experiments.

### Inter-assay consistency of measuring protection from parasitaemia following mosquito bite challenge

An endpoint that mimics clinical application is the ability of a mAb to protect from blood-stage parasitaemia following an exposure via mosquito bite challenge. In three experiments performed on different days, groups of seven mice were administered with AB311 or AB317 or human IgG isotype control AB1245. Infection by mosquitoes (*Anopheles stephensi*) carrying transgenic sporozoites occurred 16 h after antibody administration. To assess parasitaemia, blood smears were performed daily between 4- and 12-days post challenge (Table 3). Control antibody AB1245 conferred no protection in all studies. At the 600 µg dose both AB311 and AB317 conferred protection in all animals tested. Animals receiving the 300 µg dose demonstrated partial protection, and only animals receiving AB317 were protected at the 100 µg dose.

**Table 3:** Comparison of infection frequency in three protection from parasitaemia studies

mAb	Dose	Study 1		Study 2		Study 3	
		Infected	Protection (%)	Infected	Protection (%)	Infected	Protection (%)
311	100	7/7	0	7/7	0	7/7	0
	300	5/7	29	4/7	43	4/7	43
	600	0/7	100	0/7	100	0/7	100
317	100	6/7	24	7/7	0	7/7	0
	300	2/7	61	0/7	100	3/7	57
	600	0/7	100	0/7	100	0/7	100

Mice (N = 7) received either AB311 or AB317 at 100, 300 or 600 µg dose and were challenged with 5 mosquito bites post antibody administration. Appearance of blood parasites were scored from day 4 to 12 and the percent parasite free at day 12 from each group depicted

For each mAb, an ID50 was estimated using a two parameter logistic regression curve (2PL) (Table 4) to explore the differences in antibody functional activity and AB317 was tested for superior potency over AB311 based on the liver burden experiments. Compared to AB311, the ID50 for AB317 was lower for all three experiments (range 1.0- to 1.8-fold lower) indicating a trend in increased functional activity. However, a significant difference was only detected after pooling the data from all experiments (1.4-fold lower, P = 0.02).

**Table 4:** Comparison of ID50 for AB311 and AB317 in protection from parasitaemia assay

Study	AB311 (μg)	AB317 (μg)	Ratio	P value
1	341 (228, 455)	205 (119, 290)	1.7	0.07
2	304 (239, 369)	170 (*)	1.8	0.46
3	305 (229, 381)	295 (221, 368)	1.0	0.44
Pooled	320 (270, 371)	227 (178, 277)	1.4	0.02

ID50 was estimated in three studies and a pooled group with data from all three studies using 2PL analysis. * 2PL method could not estimate confidence intervals for this experiment

As with the liver burden experiments, circulating mAb serum concentrations one hour prior to challenge were measured using a CSP ELISA (Fig. 3b). IC50 was then estimated for each mAb using a 2PL model (Table 5). AB311 IC50 ranged from 61 to 87 μg/mL and the pooled estimate was 74 μg/mL. Similarly, the IC50 estimate for AB317 was lower, ranging from 41 to 59 μg, and the pooled estimate was 48 μg/mL. Similar to the ID50 comparisons, the IC50 for AB317 was consistently lower than AB311 with similar reduction range (1.0- to 1.9-fold lower). The fold reduction was statistically significant for one experiment (1.9-fold lower, P < 0.01 in Study 2) and when pooling the data across all experiments (1.52-fold lower, P = 0.02).

**Table 5:** Comparison of IC50 estimations for AB311 and AB317 in probability of infection

Study	AB311 (μg/mL)	AB317 (μg/mL)	Ratio	P value
1	87 (54, 121)	47 (27, 67)	1.9	0.06
2	78 (58, 98)	42 (30, 53)	1.9	< 0.01
3	61 (33, 90)	60 (30, 89)	1.0	0.48
Pooled	74 (57, 92)	49 (37, 61)	1.5	0.02

Using 2PL analysis, with serum antibody concentration as the input reduction of 50% of infection probability was estimated in three studies and a pooled group with data from all three studies using a 2PL model. IC50 are depicted with 95% CI in parenthesis

### Single dose comparisons using time to infection

**Fig. 4:**
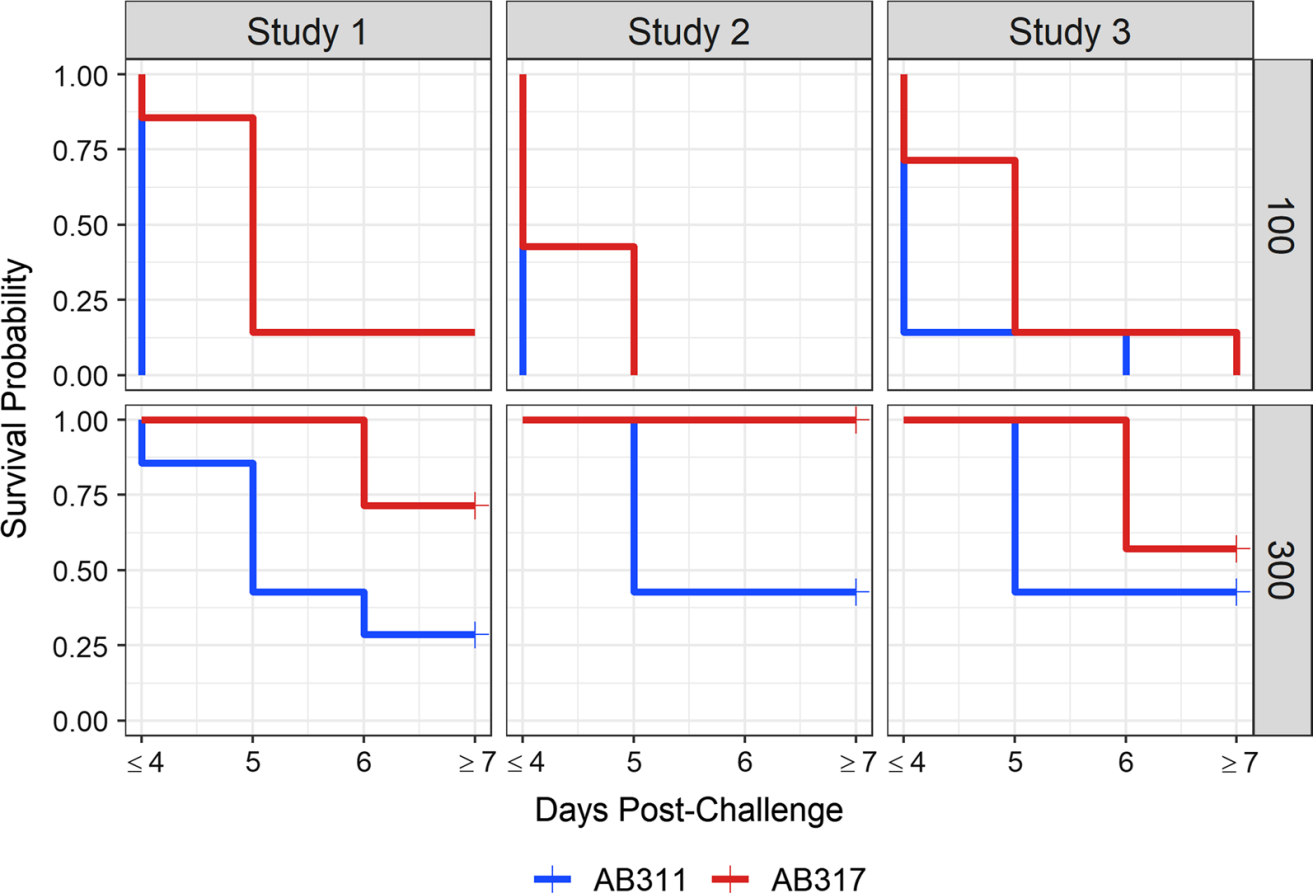
Kaplan–Meier survival curves depicting time until infection post-challenge for mice by administered mAb in protection from parasitaemia studies. For each study, mice (N = 7 each) received 100, 300, or 600 ug of either AB311 or AB317 and were monitored for parasitaemia starting 4 days post-challenge. Survival probability is defined as the fraction of animals remaining parasite free among animals at risk for each the indicated time point. Uninfected animals are denoted by right-censoring after day 7 and the final survival probability corresponds to the proportion of animals infected

Assuming that protective activity of a mAb is correlated with delay in infection, time to infection was evaluated for the ability to discriminate antibody functional activity. Using both protection and time to infection, superiority (using one-sided tests) of AB317 over AB311 was tested based on results from the liver burden experiments. For the three experiments, the 600 µg dose for both AB311 and AB317 induced full protection and identical results and, therefore, only 100 and 300 µg dose results were compared. The proportion of infected mice at the 300 µg dose was significantly lower for AB317 in one out of three studies using Barnard’s exact test (Table 6). The pooled data also showed significant difference in protection at this dose level. Next, using the Exact log rank test; significant delays in infection time for AB317 compared to AB311 were found in each study in at least one of the doses, and for pooled data at both dose levels (Table 6, Fig. 4). Of note, in Study 2 for the 100 µg dose, AB317 induced a significant delay in infection time despite 100% infected mice.

**Table 6:** Single dose comparison results between AB311 and AB317

Dose	Study	Proportion infected		Barnard P value	Log Rank P value
		AB311	AB317		
100 µg	Study 1	1.00	0.86	0.260	0.002
	Study 2	1.00	1.00	1.000	0.096
	Study 3	1.00	1.00	1.000	0.043
	Pooled	1.00	0.95	0.263	< 0.001
300 µg	Study 1	0.71	0.29	0.090	0.033
	Study 2	0.57	0.00	0.012	0.035
	Study 3	0.57	0.43	0.395	0.153
	Pooled	0.62	0.24	0.007	0.002

The proportion infected at each dose were compared using the Barnard test and survival times (Fig. 4) were compared using the log-rank test using the exact conditional distribution

### Potency comparisons across all doses

The logistic regression model was used to compare the risk of infection for mice treated with AB317 and AB311 adjusting for dose using one-sided tests (i.e., testing for superiority of AB317 over AB311). Mice dosed with AB317 had statistically significant lower odds of infection (indicating superior potency) compared to those dosed with AB311 in one experiment, were at the border of significance in one experiment, and failed to discriminate in the third experiment (Table 7). As with the comparison testing using a single dose, results were significant when the experimental data were pooled, increasing overall sample size (21 mice).

**Table 7:** Results from logistic regression model with one-sided 95% confidence interval (CI)

Study	Logistic model	
	OR	OR 95% CI upper bound
1	0.19	1
2	0.04	*0.76*
3	0.62	3.16
Pooled	0.2	*0.57*

Statistically significant comparisons (upper bound below 1) are italiciized

In the logistic regression model the odds ratio, OR, represents the odds of infection for animals in the AB317 group compared to the AB311 group adjusting for dose. In this model, an odds ratio (OR) below 1 (one) indicates superior protection for AB317 over AB311. Test for superiority were performed using one-sided 95% confidence intervals of the OR.

### Experimental design of comparison studies using both assays

**Fig. 5:**
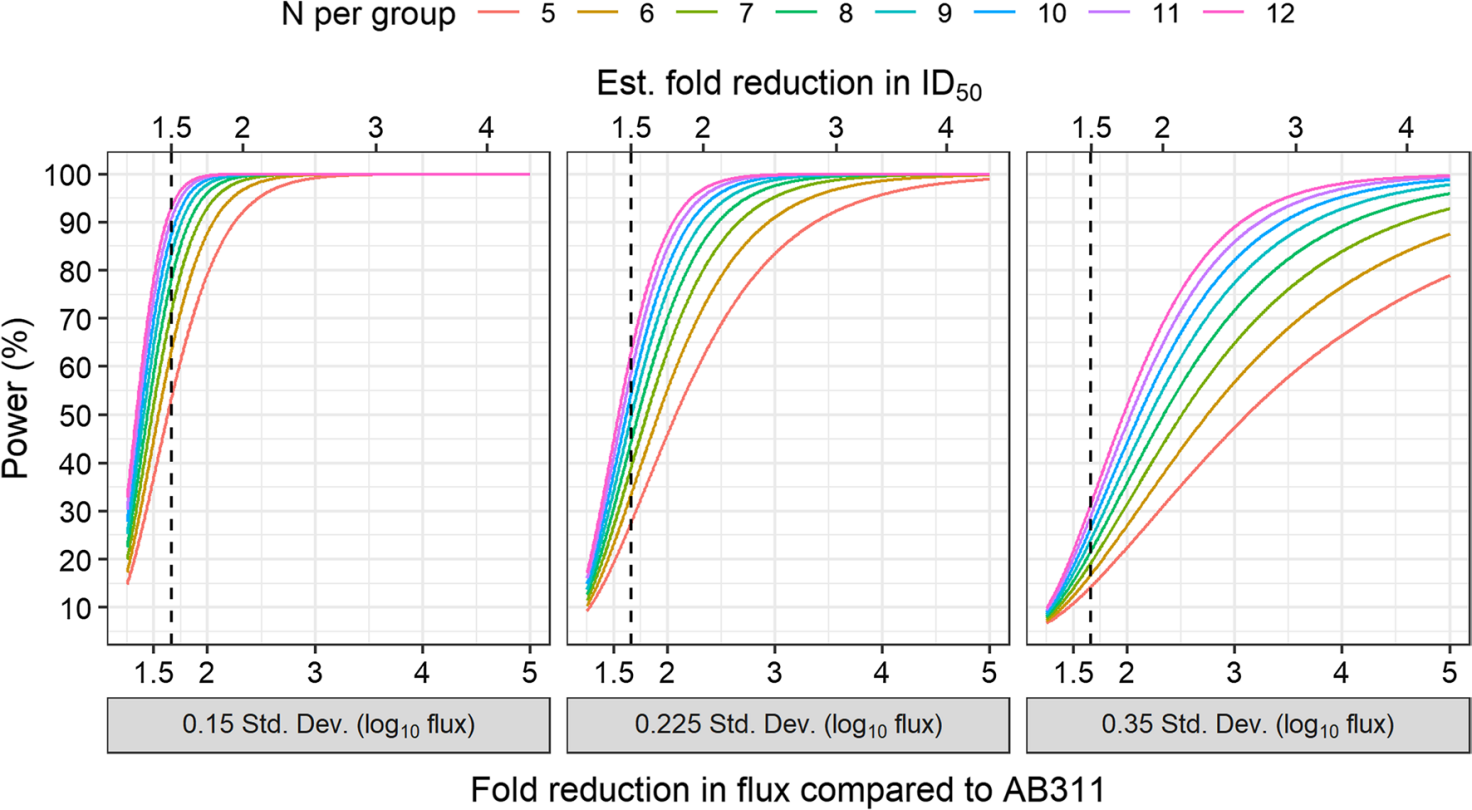
Power to detect differences in liver burden comparing a candidate mAb to AB311 administered at single dose (ID50 of AB311) for varying sample sizes and experimental variation (standard deviations of log_10_ flux). Candidate mAbs were varied by increasing potency (fold reduction in log_10_ flux compared AB311). Given a fold reduction in flux at a single dose, the fold reduction in ID50 (top axis) was estimated using the dose–response relationships (4PL models) estimated for AB317, representing a potential candidate mAb. The vertical dashed-line was the estimated fold reduction in ID50 comparing AB317 to AB311 (1.50-fold, Table 3)

The data was explored to determine how the two assays might be used in the future to identify potent mAbs among a number of candidate mAbs. For the liver burden model, the dose response curve indicates that best discrimination at a single dose is determined at the midpoint of the dynamic range, near the ID50. At the ID50, the resulting measurements are furthest from the asymptotes where the signal to noise ratio may be reduced. Using the results from this study, candidates could be compared against the AB311 reference control at a single dose near the AB311 ID50 estimated here in the range of 103–160 µg (Fig. 1). The power to detect differences will depend on the group size and the expected variation (SD) in the data obtained. Using a range of three standard deviation estimates observed in the data, the power to detect differences from AB311 was calculated across a range of potential fold flux reductions induced by the putative more potent candidate mAb (Fig. 5). A candidate mAb with similar functional activity to AB317 (1.5-fold reduction in ID50 compared to AB311) can be expected to have a 1.5- to twofold reduction in flux compared to AB311 at the given dose. Shown in Fig. 5, the power to detect such a difference in functional activity is relatively low (< 50%) when using five animals at a single dose. The current experimental designs, using five animals per group, are powered at 80% to detect candidate antibodies with 2.9-fold reduction in flux (2.4-fold ID50 change) compared to the reference control, at the average estimated standard deviation determined in our experiments (SD = 0.225). To detect antibodies that are slightly more potent (e.g., twofold reductions similar to AB317 vs. AB311) larger group sizes would be required using the assumption of average standard deviation for intra-assay variability (N = 10 for 80% power). Intra-assay variability has a large effect on discrimination: studies are only powered to detect large differences in potency using five animals per group (> fivefold flux reduction for 80% power) using the highest observed standard deviation for intra-assay variability (SD = 0.35). Furthermore, for a candidate antibody with an underlying threefold improvement in ID50, the power is > 90% using the average standard deviation but drops below 80% at the highest observed standard deviation for intra-assay variability. At this level of variation, 80% power to detect differences in ID50 of twofold is not achieved even with large group sizes (N > 12). This calculation clearly highlights the importance of maintaining good control over assay consistency when screening for antibodies with different levels of activity as exemplified by AB311 and AB317. Additionally, power might be improved by adjusting the hypothesis testing, setting non-inferiority margins, and increasing the false positive rate.

**Fig. 6:**
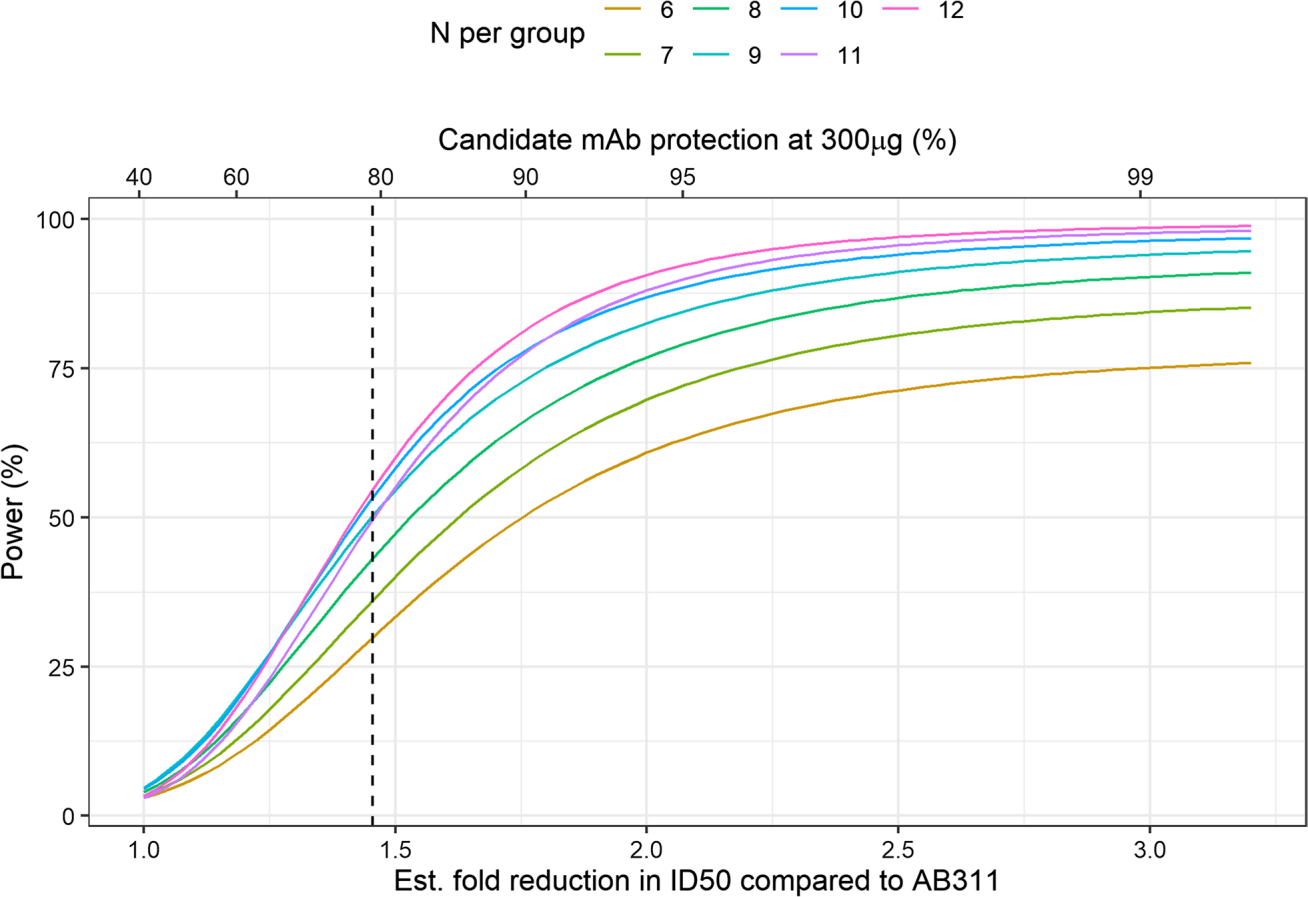
Power to detect differences in protection measured by the protection from parasitaemia assay comparing a candidate mAb to AB311 (43% protective) at a single dose (300 µg) by varying sample sizes. Candidate mAbs were varied by increasing potency interpreted as either the fold reduction in ID50 (bottom axis) or the absolute protection at 300 ug (top axis). Fold reduction in ID50 and protection at 300 ug are related through the dose–response relationships fit to AB311 and AB317 (2PL models). The vertical dashed line represents the estimated fold reduction in ID50 between AB317 and AB311 (1.45-fold) and 78% protection for AB317 at 300 µg

An analysis was also conducted to guide the testing of candidate antibodies in the protection from parasitaemia assay. Again, AB311 was considered as the reference (Fig. 6) and only the 300 µg dose data were used as there was no protection observed at the 100 µg dose. Power was calculated based on candidate antibodies with increasing protective efficacy (proportion of animals uninfected) and estimates of ID50 were approximated from protection using the logistic model’s fit to the pooled experimental data (Fig. 6). Future experiments are likely be to be designed to test mAbs for superiority, therefore one-sided tests can help improve statistical power. This analysis suggests that using the protection from parasitaemia assay to discriminate levels of functional activity is likely to be difficult unless the underlying difference in is substantial. As shown in Fig. 6, with 40% protection achieved using AB311 as a reference, a superior antibody is only detected with near 80% power when the expected protective functional activity of the candidate mAb is greater than 90% (> 1.75 reduction in ID50) and group sizes of greater than 10 are used. For experiments with a candidate similar to AB317 (1.5-fold reduction in ID50), approximately 33% power is expected using seven animals but that could be improved to around 50% using 10 animals. Similar to comparisons using liver burden reduction, power could be improved by considering non-inferiority margins or increasing the false positive rate. Additionally, experiments could be powered based on the infection time outcome as more is learned about the consistency of this endpoint through experimentation.

## Discussion

In order to identify and select novel vaccine and mAb interventions that target the CSP protein of *P. falciparum*, it is important to understand how preclinical assays can be used to measure and compare the functional potency of antibody preparations. This study analyses the consistency of results obtained in two in vivo protection assays performed in one laboratory with standardized procedures. Well-defined mAbs were used in order to standardize the input test article and it should be recognized that results described here will need to be confirmed using polyclonal sera to assess vaccine potency.

The reduction in parasite liver burden model has the advantage of providing quantitative information on the amount of liver infection while the protection from parasitaemia assay measures a more clinically relevant outcome. In both assays, challenge of mice used an engineered *P. berghei* parasite in which the natural CSP gene has been replaced by that of *P. falciparum* [34]. This challenge system may not fully replicate the desired biology of *P. falciparum* infection of the liver [35–37]; however, it can test *P. falciparum* interventions efficiently at relatively low cost in immunocompetent mice [34]. Assays using a similar approach with transgenic sporozoite expressing *Plasmodium yoelii* strain specific CSP have been published by other groups [38]. A system in which mice are rendered susceptible to *P. falciparum* infection, via engrafted human liver cells [22] may also have application to the testing of mAbs and vaccines. Direct comparisons of results for the determination of functional potency of mAbs among these model systems are warranted.

The current study strengthens comparative testing of CSP-specific mAbs by evaluating intra- and inter-assay variability. The results show these in vivo model systems can be highly reproducible. The ability to consistently prepare and deliver sporozoites for i.v. challenge is indicated by the low intra- and inter-experiment variation in log10 total flux for untreated control and mAb prophylaxis groups. The mosquito bite protection model also showed good consistency between three experiments with five infected mosquitoes causing infection in all mice in controls as well as allowing complete blockade by high doses of functional mAbs. These results indicate that infectious mosquitoes can be prepared to produce a consistent parasite challenge. Inter-assay consistency was also evident in the calculations of potency of the tested mAbs. Using the liver burden model, seven independent determinations of the ID50 for AB311 (Fig. 1) had inter-experimental overlapping confidence intervals. For AB317, consistent ID50 measurements were also determined. Similar conclusions can be drawn from the analysis of the protection from parasitaemia model with consistent ID50s calculated across three experiments for each antibody.

The results provide guidance on the ability of the assays to discriminate the relative functional activity of test articles. Multiple comparisons of AB311 and AB317 in both assays consistently showed more potency for AB317. In the liver burden assay, approximately a 1.5-fold difference in activity may be close to the limit of the assay to discriminate differences in functional activity using experimental designs reported here. This is inferred from the fact that a difference in ID50s between AB311 and AB317 could be detected (with P < 0.05) in one of two experiments and in the pooled data (Table 2). In the protection from parasitaemia assay, it was difficult to discriminate ID50 between the mAbs in all experiments and in the pooled data using only the sterile protection endpoint. Log-rank comparisons of time to patency between the two mAbs could discriminate between mAbs in most but not all experiments. Again, this suggests it may be challenging to reliably discriminate differences of the level exemplified by AB311 and AB317 in experiments with these group sizes. The results suggest that considering time to infection in the protection from parasitaemia assay as a measure of protective activity may be helpful in discriminating differences in potency; however, larger group sizes are needed given the highly significant discrimination of functional activity seen in the pooled result (Table 7).

Calculation of IC50 using circulating concentration of mAb at the time of challenge did not aid in the detection of differences in functional activity of the mAbs. Both AB311 and AB317 were produced as human IgG1, and the serum concentration was similar between the mAbs at a given dose and the 15-h timepoint monitored. It is important to recognize that similar pharmacokinetics may not occur for all mAbs tested, and consequently, measurement of serum concentration does add to the information on relative potency of mAbs compared.

The analysis of results reported here also provide guidance for how these assays can be used in the future to screen for mAbs with higher functional potency. For the parasite liver burden model, experiments can be designed to detect differences in activity exemplified by AB317 and AB311 using a single administered dose and modest group sizes of six with the important caveat that a low level of intra-assay variability is minimized. The power analysis shows that larger group sizes are required to detect differences in antibody potency when there is an increase in the observed standard deviation of measurements of infection within groups. This has important implications for establishing this assay at a new testing laboratory site.

The power analysis based on the protection from parasitaemia assay has two important implications for the design of experiments intending to identify more potent CSP antibodies. First, it will be important to identify a dose under which the control mAb (as exemplified by AB311) is consistently partially protective. Second, substantial group sizes (N > 10) will be needed to identify a more potent mAb that confers a protection increase from 40% in the reference mAb to 84% for the novel mAb (equivalent to approximately a 1.5 difference in ID50).

It is encouraging that the liver burden model and parasite protection model gave similar results when comparing the two antibodies tested. ID50 ratios between the mAbs were consistent across assays: 1.5-fold for liver burden and 1.4-fold in the protection from parasitaemia assay (comparing the pooled results (Tables 2 and 4). ID50 estimates were about threefold higher in the protection from parasitaemia compared to reduction in parasite liver burden for both mAbs. Based on these findings, it seems reasonable to expect the antibodies that perform well in reducing parasite liver burden will also perform well in a mosquito bite challenge model. Given the ease of experimentation and lower group sizes, it seems practical to screen for mAbs using the liver burden assay and confirm using the protection from parasitaemia assay. However, this relationship may not extend to antibodies that have different mechanisms of action, such as those that bind outside the NANP repeat region of CSP. A mechanistic understanding of why AB311 and AB317 display different functional activity is outside the scope of this study. It is clear that these antibodies bind differently as determined by structural studies using X-ray crystallography and by electron microscopy [16, 39]. However, the association of binding properties with functional activity remains a very active area of research and such investigations should be aided by the information in the current report.

## Conclusion

The results reported here indicate that in vivo assays measuring reduction in liver burden and protection from parasitaemia following mosquito bite challenge can provide relevant information for understanding comparative functional activity of mAbs when performed using standardized reagents and protocols. For full assay qualification and validation, which will be useful for the broader malaria research field, additional data will need to be generated and analysed. For example, in this study variation introduced by inter-laboratory or inter-personnel differences or the robustness of the assays to variation in protocol and reagents were not explored. Until such data is generated and analysed, it is recommended that standardized comparator reagents be used across laboratories; the antibodies described in this manuscript could serve this purpose. In conclusion, the results reported here indicate that these two assays can be a valuable tool to assess any newly isolated mAbs as well as to probe specific structural elements of mAbs for their impact on function. It is also anticipated that these assays will be useful as mAbs are designed and optimized for desired product characteristics for use in malaria prevention.

## Supplementary information

**Additional file 1: Table S1.** Log_10_ flux values for studies for Liver Burden studies using AB317.

## Data Availability

All data generated or analysed during this study are included in this published article.

## Abbreviations

CSP: Circumsporozoite protein
NHP: Non-human primate
RT-qPCR: Reverse transcriptase quantitative polymerase chain reaction
mAb: Monoclonal antibody
