# Supplementary material for: Characterization of two in vivo challenge models to measure functional activity of monoclonal antibodies to Plasmodium falciparum circumsporozoite protein
Source: Malar J. 2020 Mar 17;19:113. doi: 10.1186/s12936-020-03181-0 (PMC7079517; doi:10.1186/s12936-020-03181-0)
Supplement: Supplementary file 1 — Additional file 1: Table S1. Log10 flux values for studies for Liver Burden studies using AB317. [file 12936_2020_3181_MOESM1_ESM.docx]

|  |  | Study 1 | | Study 2 | |
| --- | --- | --- | --- | --- | --- |
| AB317 | Dose (µg) | Mean | SD | Mean | SD |
|  | 600 | 5.28 | 0.08 | 5.40 | 0.08 |
|  | 300 | 5.30 | 0.05 | 5.39 | 0.15 |
|  | 100 | 5.92 | 0.28 | 5.75 | 0.28 |
|  | 30 | 6.69 | 0.06 | 6.59 | 0.16 |
| Naive Infected | 0 | 7.25 | 0.06 | 7.20 | 0.15 |
| Naive Non-Infected | 0 | 5.25 | 0.02 | 5.25 | 0.03 |

Table S1 Log_10_ flux values for studies for Liver Burden studies using AB317

Individual mouse total flux values were used to calculate geometric mean for each dose group. **Abbreviations used:** mAb, monoclonal antibody; SD, standard deviation as estimated using a random effects model as described in methods.
